# Automatic extraction of gene ontology annotation and its correlation with clusters in protein networks

**DOI:** 10.1186/1471-2105-8-243

**Published:** 2007-07-10

**Authors:** Nikolai Daraselia, Anton Yuryev, Sergei Egorov, Ilya Mazo, Iaroslav Ispolatov

**Affiliations:** 1 Ariadne Genomics, Inc, 9430 Key West Ave., Suite 313, Rockville, MD 20850, USA

## Abstract

**Background:**

Uncovering cellular roles of a protein is a task of tremendous importance and complexity that requires dedicated experimental work as well as often sophisticated data mining and processing tools. Protein functions, often referred to as its annotations, are believed to manifest themselves through topology of the networks of inter-proteins interactions. In particular, there is a growing body of evidence that proteins performing the same function are more likely to interact with each other than with proteins with other functions. However, since functional annotation and protein network topology are often studied separately, the direct relationship between them has not been comprehensively demonstrated. In addition to having the general biological significance, such demonstration would further validate the data extraction and processing methods used to compose protein annotation and protein-protein interactions datasets.

**Results:**

We developed a method for automatic extraction of protein functional annotation from scientific text based on the Natural Language Processing (NLP) technology. For the protein annotation extracted from the entire PubMed, we evaluated the precision and recall rates, and compared the performance of the automatic extraction technology to that of manual curation used in public Gene Ontology (GO) annotation. In the second part of our presentation, we reported a large-scale investigation into the correspondence between communities in the literature-based protein networks and GO annotation groups of functionally related proteins. We found a comprehensive two-way match: proteins within biological annotation groups form significantly denser linked network clusters than expected by chance and, conversely, densely linked network communities exhibit a pronounced non-random overlap with GO groups. We also expanded the publicly available GO biological process annotation using the relations extracted by our NLP technology. An increase in the number and size of GO groups without any noticeable decrease of the link density within the groups indicated that this expansion significantly broadens the public GO annotation without diluting its quality. We revealed that functional GO annotation correlates mostly with clustering in a physical interaction protein network, while its overlap with indirect regulatory network communities is two to three times smaller.

**Conclusion:**

Protein functional annotations extracted by the NLP technology expand and enrich the existing GO annotation system. The GO functional modularity correlates mostly with the clustering in the physical interaction network, suggesting that the essential role of structural organization maintained by these interactions. Reciprocally, clustering of proteins in physical interaction networks can serve as an evidence for their functional similarity.

## Background

Modularity in biological networks was proposed more than a decade ago as a way for a cell to organize functional blocks and maintain specificity of cellular regulation [1]. Since then, numerous attempts to detect modules in biological networks have been described [2-4]. In many of these studies, the Gene Ontology [5] (GO) has been used as the "gold standard" to validate the functional relevance of the found network clusters [6,7]. Being the *de facto *international standard for protein functional annotation, GO constantly evolves, changing both classification hierarchy and description of individual proteins. Structurally, GO is a directed acyclic graph of terms (nodes) connected with links representing two types of term relations: "is-a" and "part-of." GO has three major branches covering corresponding aspects of protein functions: biological process, molecular function, and cellular components. The approaches for assigning GO terms to proteins can be grouped in two major classes.

The first class uses predictive methods to transfer existing annotation to new or experimentally uncharacterized proteins. These methods utilize different learning techniques (Bayesian networks, decision trees, rule learning, etc.) to assign GO annotation (GOA) based on: i) similarity of sequence or sequence features, including amino acid and domain composition of proteins and 3D structure [8-16], ii) similarity of expression profiles derived from high-throughput microarray experiments [17-21], and iii) analysis of protein interaction networks [22,23].

The second class of approaches determines protein's function by dedicated experiments. The experimental facts, describing additional functions of known proteins as well as roles of the uncharacterized proteins, are collected and systematized by reading and analyzing scientific literature. The sheer number and the rate of production of scientific publications make a comprehensive manual analysis of scientific literature nearly impossible [24]. Therefore, various text-processing techniques for automatic extraction of protein function information from scientific publications have been proposed. Raychaudhuri *et al. *[25] used a two-step approach: assigning GO terms to PubMed abstracts based on the abstracts' similarity and a training set of pre-annotated abstracts, and then assigning identified GO terms to proteins based on statistical analysis of their occurrences in PubMed abstracts. Chiang *et al. *[26] proposed another technique, in which GO terms were first detected in text, and then GO-to-protein associations were extracted by unsupervised learning of linguistic patterns from a training set and employing Bayesian statistics. A more sophisticated approach is described by Koike *et al. *[27], in which GO terms were detected in raw text and, by using shallow parsing techniques, protein-GO associations were extracted. This approach also included extensive augmentation of GO terms prior to the detection step.

So far, it has not been comprehensively demonstrated whether these two approaches to annotate protein with GO terms yield mutually consistent results. The purpose of our study is to obtain such cross-validation and to establish the accuracy and credibility of the machine-based assignment of protein function. Reciprocally, we want to confirm conclusions about protein function drawn from microarray experiments and protein network analysis that use the GO annotation. Our paper first describes a new, fully automated method for literature-based annotation of proteins with "biological process" GO terms and its consistency with existing GO annotation. Other two GO branches "Cellular component" and "Molecular function" have smaller number of groups and can be annotated with high accuracy by methods based on sequence homology. Second, we show a match between the GO groups and putative functional complexes, determined as clusters in protein networks.

In the first part of our work, we developed advanced linguistic tools for detecting GO terms in text and extracting protein-GO associations with accuracy more than 90% compared to the manual text processing. When applied to the entire PubMed database, it has extracted more than 400,000 individual protein-GO associations. We present the results of a comparison of the extracted annotation to the existing public annotations that supports the validity of the former one. To validate our NLP approach, we uncover a significant overlap between the newly obtained and existing GO annotations and also discuss possible reasons for discrepancies between them. In the second part of our work we demonstrate that proteins belonging to a GO group are much more likely to interact with each other than expected by pure chance (in random network re-wiring). The observed correlation is further confirmed by the complementary observation that virtually every densely linked network community has a strongly non-random overlap with one or several corresponding GO groups.

## Results

### Evaluation of protein-GO association extracted by MedScan technology

The extension of the MedScan natural processing technology to detect GO terms and protein-GO association is described in the Methods section and in Additional file 1. We have applied our GO term matching algorithm to the entire 2004 release of Medline and have identified 3.3 million instances that represent 3,562 unique "Biological process" GO terms. Interestingly, 380 of the most frequent GO terms are responsible for 90% of all matches, while 1,128 GO terms were responsible for 98% of all matches. We have manually inspected several thousand randomly selected matches and determined that GO terms are identified with an accuracy above 98%.

We have obtained 407,044 individual protein-GO associations from Medline 2004 release using the existing MedScan dictionary for mammalian proteins and new GO term dictionary described in the Methods section. Because many associations were extracted multiple times, we have compressed them into 72,390 unique protein-GO associations for 8,400 mammalian proteins. After compression, we assigned each association a reference count indicating the number of times this association was extracted. 62% of associations had only one reference; 10.6% associations had more than five references.

To estimate the accuracy of protein-GO association extraction, we have randomly selected 193 sentences, which contain at least one pair of a protein and a GO term and manually extracted 107 protein-GO associations from them. MedScan extracted 94 protein-GO associations from the same sentences. Upon comparison with manually extracted associations, we found that only nine relations were incorrect. This limited evaluation put the precision of the automatic extraction at 90.4% and recall at 79.4%. Hence the standard F-score for our extraction method calculated as 2⋅presision⋅recallpresision+recall
 MathType@MTEF@5@5@+=feaafiart1ev1aaatCvAUfKttLearuWrP9MDH5MBPbIqV92AaeXatLxBI9gBaebbnrfifHhDYfgasaacH8akY=wiFfYdH8Gipec8Eeeu0xXdbba9frFj0=OqFfea0dXdd9vqai=hGuQ8kuc9pgc9s8qqaq=dirpe0xb9q8qiLsFr0=vr0=vr0dc8meaabaqaciaacaGaaeqabaqabeGadaaakeaabaWaaSaaaeaacqqGYaGmcqGHflY1cqWGWbaCcqWGYbGCcqWGLbqzcqWGZbWCcqWGPbqAcqWGZbWCcqWGPbqAcqWGVbWBcqWGUbGBcqGHflY1cqWGYbGCcqWGLbqzcqWGJbWycqWGHbqycqWGSbaBcqWGSbaBaeaacqWGWbaCcqWGYbGCcqWGLbqzcqWGZbWCcqWGPbqAcqWGZbWCcqWGPbqAcqWGVbWBcqWGUbGBcqWGRaWkcqWGYbGCcqWGLbqzcqWGJbWycqWGHbqycqWGSbaBcqWGSbaBaaaaaaa@5C65@ is equal to 0.85. To better measure the accuracy of MedScan extraction we have manually counted the false positives in two random samples of associations extracted by MedScan. First sample had 13.6% false positive rate (41 out of 301 associations), second sample had 15.3% false positive rate (46 out of 300 associations). This made the average MedScan precision 87.2 ± 2.9 %.

**Figure 1:**
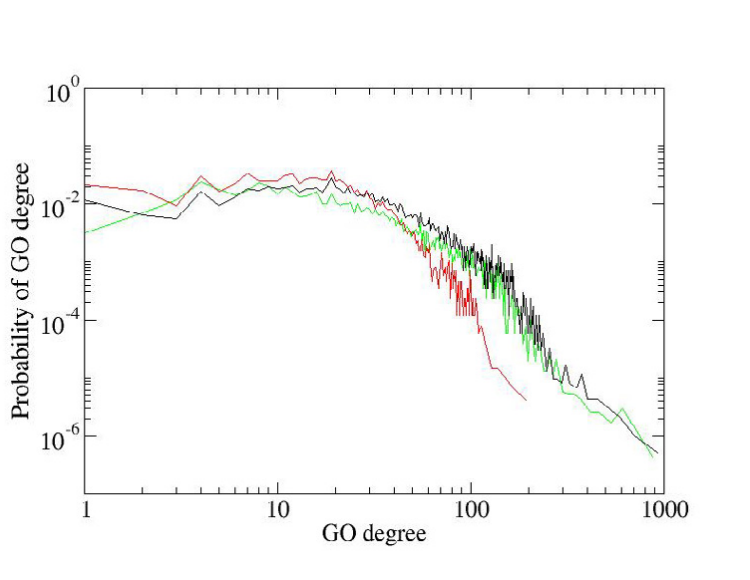
Distribution of the number of protein-GO association for three GO annotations. Horizontal axis, GO degree – number of GO associations; Vertical axis, Probability of GO degree – fraction of proteins with a given GO degree; Red line – public GOA, green – MedScan GOA, black – combined GOA.

We then compared the entire set of extracted annotations with the complete publicly available annotation for mammalian proteins. GO annotations for human, mouse, and rat provided by EBI was obtained from the Gene Ontology Website and combined together. For comparison with our extracted annotation, we retained only annotations representing the "Biological process" branch of the ontology. We have mapped 69,255 out of 100,478 protein annotation records from public annotation to Entrez Gene identifiers that are also used by MedScan. We found that the majority of the remaining 31,223 unmapped associations belonged to unverified protein records (TREMBL, predicted transcripts, etc.). The 69,225 annotation records were further reduced to 44,254 unique [protein ID, GO ID, evidence code] triplets. To compare public and MedScan annotation, we expanded both GOAs by adding every parent GO group of the original annotation to a protein. We found that 78,346 protein-GO associations were identical between expanded public and MedScan GO annotations. This constituted 20% of public expanded annotation and 24% of MedScan expanded annotation. We also determined that 6,986 proteins had at least one identical annotation and on average each protein had 43% of annotations identical between public and MedScan GOAs. The expanded public annotation had on average twenty GO groups per protein covering 19,946 proteins, while MedScan GOA had 39 GO groups per protein covering 8,246 proteins. The distributions of the GO annotations among proteins for public, MedScan, and combined GOAs are shown in Figure 1.

Significant portion of public GO annotation was created by human curators reading scientific articles. This allowed us to compare MedScan performance with results of human annotation using the same literature corpus. We have identified 2,864 references that were used by EBI curators to produce 14,544 protein-GO group associations annotating 6.694 proteins with 1,977 biological processes. MedScan has extracted 8,437 protein-GO group associations from the same abstracts annotating 2,655 proteins with 590 biological processes. The underperformance of MedScan is easy to explain because EBI curators used full-text articles while MedScan extracted annotation only from the abstract text. We have focused further comparison only on 512 GO groups that were recognized by both MedScan and EBI curators. This allowed us to correct for the absence of full article text during MedScan extraction as well as for the absence of some GO groups in MedScan dictionaries. We found that MedScan has extracted 4,649 unique protein-GO associations, while EBI curators extracted 1,138 protein-GO associations for the same set of GO groups. Thus, EBI curators extracted three times less protein-GO associations than MedScan did from the same set of articles and GO groups. This number surprises even more if we remember that EBI curators read the entire article while MedScan used only abstract text for extraction. To prove that human curators miss many true protein-GO associations we have identified 317 protein-GO associations that were identical between EBI and MedScan annotations and were extracted from the same set of 2,864 articles by both approaches. Again, 821 associations missing from MedScan annotation among 1,138 associations extracted by curators can be attributed in large to the fact that MedScan extracted information from abstract text only. Surprisingly however, only 180 out of 317 identical associations were derived from the same article by both MedScan and curator. Thus, EBI curators missed at least 137 true protein-GO associations when reading the 2,864 articles.

The comparison with EBI manual curation has also allowed us to compare MedScan with other NLP methods for automatic extraction of protein-GO associations that were evaluated in BioCreAtIvE task 2.2. According to the original article "*the purpose of sub-task 2.2 resembled the typical human annotation procedure, in the sense that the participants had to return the annotations derivable from a given protein-article pair. The annotations which are contained within the articles should thus be automatically identified and the corresponding GO-term returned together with the supporting text passage*"[28]. Assuming the worst case scenario the 137 true protein-GO associations were not simply missed but rejected by EBI curators. For example, they could have dismissed ambiguous and hypothetical statements that MedScan interpreted as true positives (see discussion section for more details about MedScan sources of errors). In this case, we can add MedScan performance results to table 5 from Blaschke et al [28] using the numbers from the aforementioned comparison with EBI curation:

*Participant*: Daraselia et al.; *Run*: 1; *Evaluated results*: 317; *Perfect prediction*: 180 (56.8%).

**Figure 2:**
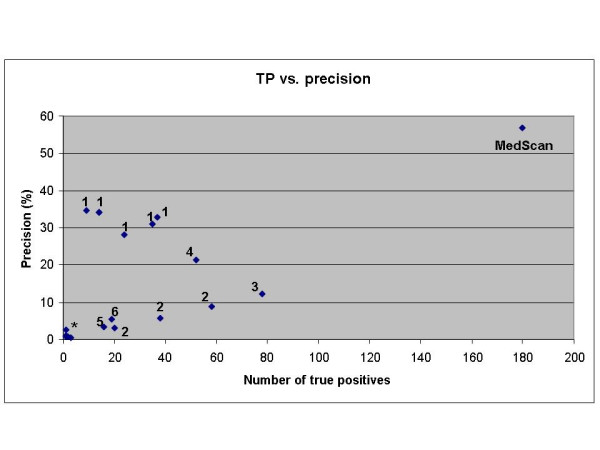
Comparison of MedScan performance with other methods for automatic protein annotation reported in Blaschke et al [28] for BioCreAtIvE task 2.2. The figure is copied from Figure 4 of Blaschke et al [28] and MedScan performance added as one more method. Each point represents a single run submitted by the participants of task 2.2. User 1: Chiang et al. [45], 2: Couto et al. [46], 3: Ehrler et al. [47], 4: Ray et al. [48], 5: Rice et al. [49], 6: Verspoor et al. [50]. MedScan performance was estimated by comparison with the protein-GO annotation extracted by human curators from European Bioinformatics Institute (EBI).

Because these numbers assume the worst case scenario they give the lower estimate for MedScan performance relative to other NLP methods. Using this numbers we position MedScan relative to other methods for automatic GO annotation on Figure 2.

Measuring identical records, however, is not adequate for comparing two hierarchical annotations due to ambiguity in the annotation process (see the Discussion section for more details). We therefore developed a hierarchical evaluation measure as described in the Methods section. This method evaluates the similarity between two hierarchical annotations by a score ranging between zero and one. Following the guidelines provided by the GO Consortium, 44,254 unique GO annotation records were split in three groups, reflecting the quality of annotation according to their evidence code:

1) A high-confidence group containing the manual annotation with evidence codes TAS, IDA, and IC;

2) An average-confidence group containing mainly the annotation derived from high-throughput experiments with evidence codes IMP, IGI, IPI, ISS, IEP, and NAS;

3) A low-confidence group containing sequence-similarity-based "electronic" annotation with evidence codes IEA, ND, and NR.

Table 1 shows hierarchical similarities among the three classes of public annotation and to the MedScan annotation. In addition, we have measured the similarity of all annotations to a randomized annotation in order to estimate the statistical significance of the similarities. We found that all annotations, including the annotation generated by MedScan, are similar to each other with a score of 0.51 on average, while the average similarity score to randomized GOA was 0.275. This result is discussed further in the Discussion section.

**Table 1:** Similarities of the three classes of GO public annotation to each other and to the GO annotation extracted by MedScan.

Annotations	High confidence GOA	Average confidence GOA	Low confidence GOA	MedScan GOA
High confidence GOA	1.0	0.46	0.52	0.52
Average confidence GOA	0.46	1.0	0.48	0.52
Low confidence GOA	0.53	0.45	1.0	0.51
Randomized GOA	0.25	0.23	0.27	0.35

The GO annotation classes are described in the Results section.

### Construction of pathways using GO annotations

To investigate network clustering within the functional modules and to further validate MedScan GO annotation, we have constructed several collections of pathways for three GOAs: public biological processes GOA (public GOA), MedScan biological processes GOA (MedScan GOA), biological processes GOA combining MedScan and public annotation (combined GOA). A pathway contains only those proteins from a GO group that interact with each other; the protein-protein interactions were taken from the ResNet 4.0 database described previously [29]. Further details of pathway composition procedure can be found in the Methods section. The manual inspection of pathways built from Biological processes GOAs showed that interactions used for their construction are relevant to the corresponding biological processes. The interactions describe the formation of protein complexes involved in a biological process, as well as regulatory events between complexes and upstream regulators such as cytokines or transcription factors. The cytokines also appear as downstream molecules regulated as a result of a biological process.

More than half of GO groups did not contain any proteins. Upon inspection we found that the majority of empty GO groups were irrelevant for the mammalian species. Because we have used only the mammalian protein names dictionary for extraction of protein-GO associations, while GO terms have been identified in the text regardless organism specificity, we believe that this observation provides additional support for the validity of annotations extracted by MedScan.

Summary of the pathways' characteristics is shown in Table 2. About 65% of GO groups containing more than one protein produced a pathway in all three GOAs. We found that the MedScan GOA allows construction of about the same number of pathways as the public GOA. However, the number of pathways increased 1.5 times when these two GO annotations were combined. This number indicates that MedScan GOA adds functionally relevant proteins to the public GO groups, and allows the construction of higher number of biological processes pathways. The utility of these pathways for analysis of the high-throughput biological data is detailed in the Discussion section.

**Table 2:** Number of biological processes pathways constructed using different GO annotations

Annotation	Number of proteins	Number of connected proteins	Number of groups with at least one protein/two proteins	Number of pathways
Biological process GOA (public)	19,460	8,272	4,194/3,280	1,994
Biological process GOA (MedScan)	8,246	6,303	3,102/2,602	1,862
Biological process GOA (combined)	20,722	8,963	5,210/4,251	2,858

Pathways were constructed as described in the Methods section. The Public Biological process GOA is publicly available GO annotation; MedScan GOA – GO annotation obtained by MedScan technology, combined GOA – GO annotation combined from public and MedScan annotations; Cellular component GOA and Molecular function GO. The number of connected proteins is the number of proteins with at least one relation to another protein in the same pathway. The number of groups is the number of GO groups that contain not less than the specified number of proteins not necessarily connected to each other. The number of pathways is the number of such GO groups that contain at least two proteins connected to each other by one or more relations from the ResNet 4.0 database.

### Higher-than-average number of protein interactions within GO annotations

**Figure 3:**
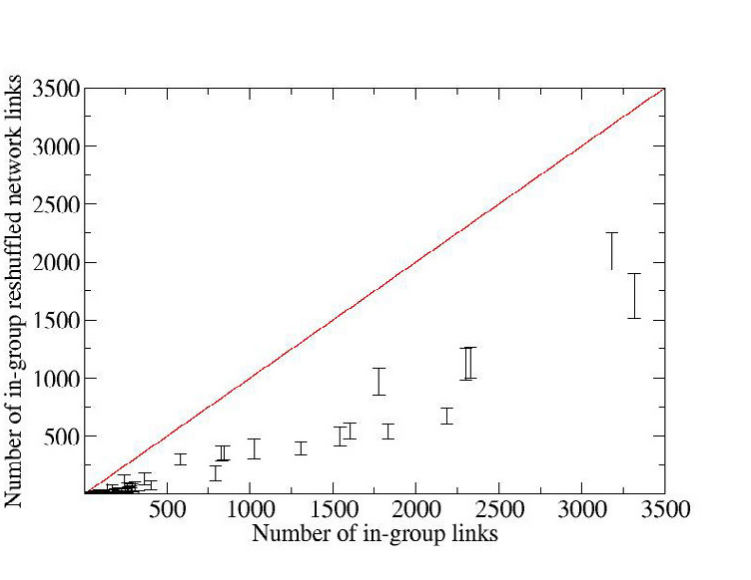
A scatter plot of the number of links of a randomized versus real binding network in the public cellular component GOA. All GO groups below the diagonal line have the number of randomized links lower the real ones. The error bars correspond to the p-value 10^-6 ^for normal distribution; that is, if the top of an error bar lies below the diagonal line, the probability that the corresponding GO group has this number of links by pure chance is equal or less than 10^-6^. It appears that only a few small GO groups are not linked densely enough to satisfy the 10^-6 ^threshold.

To check the hypothesis that cellular functional modularity is achieved by the increased link density in the molecular interaction network and to further study MedScan extraction accuracy, we then investigated whether proteins within a GO group had an increased probability to interact with each other than with arbitrary network proteins. Since the experimentally obtained physical interaction networks have been already shown to cluster within GO groups, we have analyzed the interaction networks extracted from the literature. Networks of both physical and regulatory protein interactions were taken from our ResNet 4.0 database, described previously [29]. Unlike pathways described in the previous section, the networks contained all possible relations of only one type between proteins in a GO group. In addition to three Biological process GOAs described above, we analyzed two other publicly available annotations, Cellular Components and Molecular Function. We have compared the number of links of each type between proteins within each GO group to the number of links spanning the same set of proteins in the randomized network. The degree-preserving network randomization was accomplished by reconnecting pairs of links, avoiding the steps leading to double links and self-interactions [30]. The results of our analysis for six types of protein networks, corresponding to different relation types in ResNet 4.0, and for five Gene Ontology branches are illustrated in Figure 3 and summarized in Table 3. Our main conclusion is that the large fractions of GO groups have the link density that is significantly higher than expected by pure chance. This indicates a strong correlation between functional annotation and network link density, i.e., clustering, in ResNet 4.0 (Table 3).

**Table 3:** Correlation between GO functional annotation and link density in networks from ResNet 4.0 database

**Network Annotation**	ResNet 4.0	Physical	Regulation	Expression	Molecular Transport	Promoter Binding
Biological process GOA (public)	**62.9% **(1247/1982)	**64.1% **(1247/1946)	**48.0% **(909/1895)	**28.8% **(527/1833)	**25.7% **(383/1491)	**8.3% **(103/1234)
Biological process GOA (MedScan)	**41.8% **(777/1858)	**56.2% **(1026/1825)	**14.2% **(262/1851)	**7.4% **(136/1836)	**22.6% **(389/1722)	**7.6% **(121/1595)
Biological process GOA (combined)	**47.6% **(1363/2861)	**59.2% **(1662/2809)	**21.5% **(595/2762)	**13.8% **(376/2722)	**21.6% **(524/2428)	**6.6% **(142/2166)
Cellular component GOA (public)	**80.9% **(361/446)	**78.7% **(351/446)	**43.0% **(165/384)	**34.6% **(128/370)	**43.1% **(118/274)	**1.0% **(2/209)
Molecular function GOA (public)	**56.6% **(489/864)	**53.3% **(434/815)	**35.1% **(269/767)	**27.3% **(196/719)	**19.5% **(99/507)	**2.5% **(9/357)

Table 3 shows the percentage of GO subnetworks with high link density for six network types in ResNet 4.0 for five GOAs. A GO sub-network was created by connecting all proteins within a GO group by all possible links from the given network type. All proteins that form child groups were included into the parent group recursively. The numbers in bold face indicate the fraction of these subnetworks that are **densely linked**, while the numbers in brackets show how this fraction was calculated. The first number in brackets indicates the total number of densely linked sub-networks and the second number shows the total number of all GO sub-networks. ResNet 4.0 contains *Physical *interaction relations (*Binding + Protein Modification*) and regulatory relations (*Regulation, Expression, MolTransport, PromoterBinding*). The statistics for all networks was described previously [29] and is summarized in Table 5. *Regulation *network was considered without *DirectRegulation *relations indicating the regulation by means of physical interaction.

To compute the number of densely linked GO sub-networks, we have randomized corresponding network using the algorithm that preserves the number of interaction partners of each protein [30]. After 10 randomizations, we calculated the average number of relations and their standard deviation in every GO sub-network and compared them to the number of the original network relations in every GO group. We then counted number of GO groups for which the number of actual links is greater than the number of randomized links by at least five standard deviations. Assuming that the randomized number of links in a GO group is normally distributed, this means that the selected GO groups could have such elevated number of links by pure chance only with a *p*-value less than 10^-6^.

We also found that the addition of MedScan GOA to the public GOA did not affect the fraction of the GO groups that are densely linked, keeping it around 60% for the physical interaction network. Thus, the addition of MedScan GOA does not affect the average quality of GO groups as judged by the physical interaction link density. This further validates the GO annotation extracted by MedScan.

### Fraction of densely linked GO groups is higher in physical interaction network than in regulatory network

We investigated how the GO functional annotation modules clustered in each of the five types of relations in ResNet 4.0 and for ResNet 4.0 as a whole. The interactions in ResNet 4.0 can be divided into direct physical (binding and protein modification) and the remaining indirect regulatory links. Table 3 shows that the GO groups correlate best with clusters from the direct physical interaction network. The *Regulation *network has on average 2.2 times fewer GO groups with high link density than the *Physical *interaction network. For example, in the biological process GOA obtained by combining public and MedScan annotations, 59.2% of the GO groups had a number of relations significantly higher than expected in the randomized physical interaction network. For the *Regulation *relations this number was only 21.5%. The number of statistically significant densely linked GO groups is even smaller for the other types (*Expression, Molecular transport, and Promoter Binding*) of regulatory networks.

**Figure 4:**
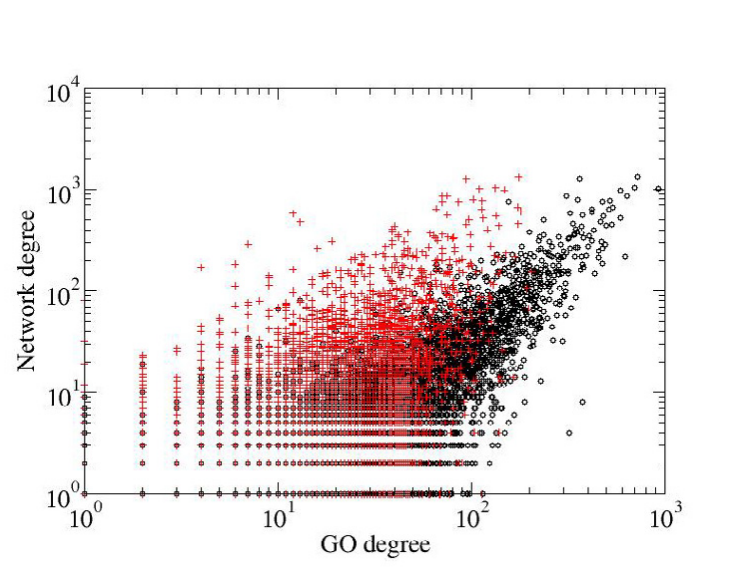
Dependence of protein degree in ResNet 4.0 (i.e. the number of regulatory interactions with other proteins in the database) on the GO degree (i.e. number of GO annotation for a protein). Red pluses – public Biological processes GO annotation; black circles – combined public and MedScan GOA. The plot shows that MedScan GOA adds highly connected proteins to GO groups from public annotation.

Even more, the number of public GO groups with significantly higher *Regulation *link density is almost three times higher than that of the MedScan (Table 3). As a consequence, the combined public and MedScan GO annotation also shows the reduced correlation with link density in the *Regulation *network. This finding can be explained by the observation that MedScan annotates highly cited proteins with many different GO groups. The highly cited proteins, such as insulin, MAPK1 or p53, are often hubs in the regulatory network, being linked to many different proteins including those in the GO groups different from its own. This effectively reduces the relative number of in-group links and, conversely, increases the connectivity between the groups. To further support this explanation, we found that the top 100 most connected proteins are present in 51% of all pathways built from the public GOA, in 88% of all pathways built from MedScan GOA, and in 77% of all pathways built from the combined GOAs. That MedScan adds highly connected proteins to GO groups is clearly visible in Figure 4, showing how the number of GO annotations for a protein depends on the number of its regulatory interacting partners. The regulatory hubs, however, are not necessarily hubs in the physical interaction network and therefore do not reduce the density of physical links in the MedScan GO groups. This effect is exemplified by hormones and cytokines that physically interact only with the corresponding receptors or extracellular carrier proteins yet regulate or are being regulated by many biological processes and many proteins.

### A comprehensive match between network clusters and functional annotation

**Figure 5:**
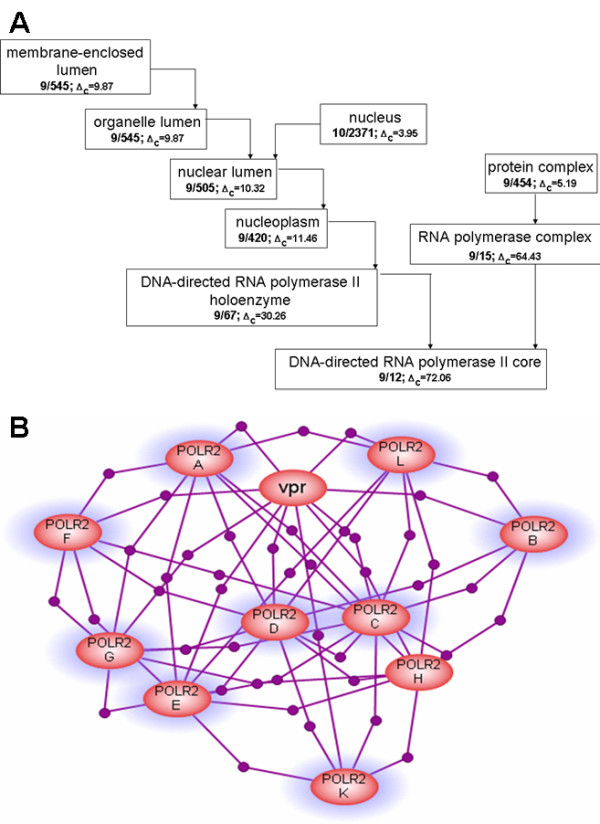
An overlap between a network cluster obtained by the Potts model algorithm [31] and the best-matching GO groups from the public cellular component GOA. The cluster contains 11 proteins: 10 subunits of RNA polymerase II and a *Vpr *protein from Human immunodeficiency virus 1. RNA polymerase II is a well-characterized and stable multi-subunit complex that is formed due to the physical interactions of its subunits. RNA polymerase II is involved in the mRNA synthesis for all eukaryotic protein-coding genes. *Vpr *protein from HIV has diverse function and regulates the expression of many cellular genes during HIV infection as well as accelerates the production of viral proteins. **A **– The portion of GO classification overlapping with network cluster. The figure shows the part of the GO classification hierarchy with the bottom node being the GO group that has the statistically the best overlap with the Potts cluster. GO groups are depicted as rectangles and the parent-child relation in the GO tree is shown as a line with an arrow. Only those parent GO groups that have a statistically significant overlap with the Potts cluster are shown. The numbers above the line show the number of proteins common with the Potts cluster (before the slash) and the total number of proteins in the GO group. The Δ_c _value below the arrow is the number of standard deviations by which the overlap is bigger than the overlap expected by random chance. B – The network cluster overlapping with GO classification from Figure A. Highlighted proteins belong to the best overlapping GO group from cellular component classification DNA-directed RNA polymerase II, core complex (GO:0005665).

**Figure 6:**
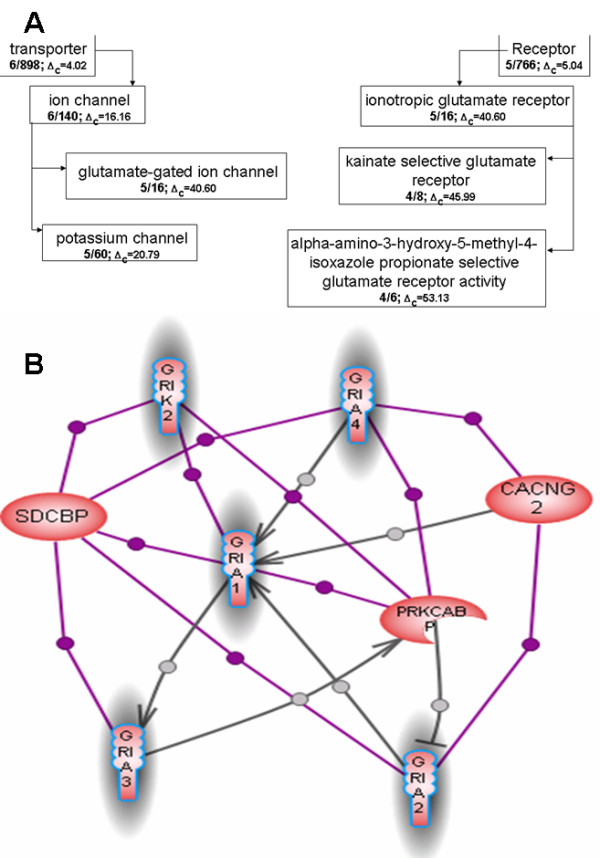
An overlap between a network cluster that was obtained by the Potts algorithm [31] and the best matching GO groups from the molecular function GOA. The cluster contains eight proteins: five heterodimerizing proteins from the ionotropic glutamate receptor family, syndecan binding protein SDCBP, gamma subunit 2 of voltage-dependent calcium channel (CACNG2), and protein kinase C alpha binding protein (PRKCABP). The molecular function GOA shows the smallest correlation with network clustering among all GOAs (see Results section for details). Nevertheless, the correlation is still significant and provides additional confirmation to the observation that paralogous proteins tend to interact with each other more often than non-paralogous proteins [39]. The picture shows the example of the paralog heterodimerization that form a cluster in the physical interaction network. **A **– The portion of GO classification overlapping with network cluster. The GO classification tree depiction is the same as in Figure 5A. **B **– The network cluster overlapping with GO classification from Figure A. Highlighted proteins belong to the best overlapping GO group from molecular function classification – alpha-amino-3-hydroxy-5-methyl-4-isoxazole propionate selective glutamate receptor activity (GO: 0004971). The proteins selected by the blue line belong to the second best overlapping GO group from molecular function classification – potassium channel activity (GO:0005267). Gray links indicate *DirectRegulation *relation, violet links indicate *Binding *relation.

**Figure 7:**
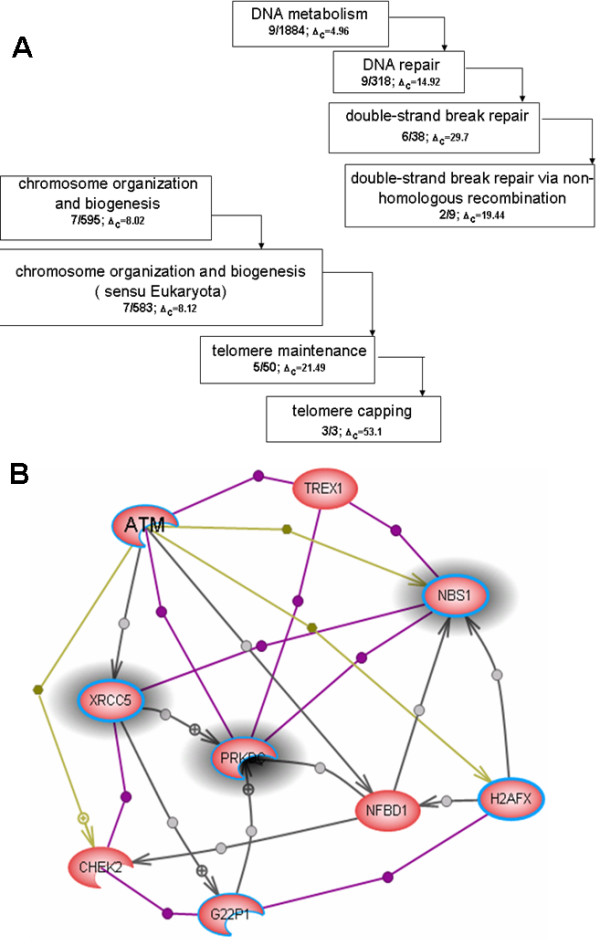
An overlap between a network cluster obtained by Potts algorithm [31] and the best matching GO groups from the biological function GOA combined from MedScan annotation and public annotation. The cluster contains nine proteins involved in DNA repair and telomere capping: ATM – ataxia telangiectasia mutated homolog (human) (mapped); PRKDC – catalytic polypeptide of DNA activated protein kinase; NBS1 – nibrin; CHEK2 – protein kinase Chk2; XRCC5 – X-ray repair complementing defective repair in Chinese hamster cells 5; H2AFX – dolichyl-phosphate (UDP-N-acetylglucosamine) N-acetylglucosaminephosphotransferase 1 (GlcNAc-1-P transferase); G22P1 – thyroid autoantigen; NFBD1 – mediator of DNA damage checkpoint 1; TREX1 – three prime repair exonuclease 1. The ataxia-telangiectasia mutated (ATM) kinase signals the presence of DNA double-strand breaks in mammalian cells by phosphorylating proteins that initiate cell-cycle arrest, apoptosis, and DNA repair. The Mre11-Rad50-Nbs1 (MRN) complex acts as a double-strand break sensor for ATM and recruits ATM to broken DNA molecules [42]. Activated ATM phosphorylates its downstream cellular targets H2AFX and Chk2 as well as proteins directly involved in DNA repair: XRCC5, TREX1 and NFBD1. G22P1 and PRKDC are subunits of DNA activated protein kinase that can be induced by DNA damage to promote DNA end joining [43]. It also can attenuate CHK2 control of the damage checkpoint [44]. **A **– The portion of GO classification overlapping with network cluster. The GO classification tree depiction is the same as in Figure 5A. **B **– The network cluster overlapping with GO classification from Figure A. Highlighted proteins belong to the best overlapping GO group from molecular function classification – telomere capping (GO:0016233). The proteins selected by the blue line belong to the second best overlapping GO group from combined biological processes classification – double-strand break repair (GO:0006302). Gray links indicate *DirectRegulation *relation, violet links indicate *Binding *relation, and green arrows represent *ProtModification *relations.

To further support our claim that GO groups are usually strongly intralinked, we looked at the correspondence between the densely linked network clusters and GO pathways from the other direction. We studied how well the densely linked *Binding *network clusters, obtained using solely the network topology information, matched with the GO groups. The network clusters are produced using an annealing in the network Potts model (see Methods and [31,32]). A typical single annealing run on the ResNet binding network produces 40-100 densely linked communities, each consisting of 8-80 proteins. We found a perfect overlap between more than 90% of all found network communities and corresponding GO groups, or often whole hierarchical trees of them (Table 4). The examples of strong correspondence between network clusters and functional annotation are shown in Figures 5, 6, 7.

**Table 4:** Overlap between clusters in physical interaction network and GO annotation

Biological process (public GOA)	Biological process (MedScan GOA)	Biological process (combined GOA)	Cellular component (public GOA)	Molecular function (public GOA)
54	50	54	49	54

A total of 54 network clusters, each containing between 8 and 82 proteins were obtained in the given realization of Potts network clustering method [31]. The statistical significance of the overlap between every cluster and every GO group of a given annotation was evaluated using Fisher's Exact Test by computing the probability that the cluster and a GO group, drawn from the same set of ~10000 proteins at random, have the overlap not less than the observed one. The table shows the number of network clusters overlapping with at least one GO group in a given annotation with a *p*-value less than 0.001.

**Table 5:** ResNet 4.0 networks statistics

**Name**	**Count**
Number of proteins with a link of any kind	10,739
*Binding *links	30,448
*Regulation *links	58,263
*Expression *links	24,293
*MolTransport *links	5,481
*PromoterBinding *links	3,201

The number of proteins and various types of relations between proteins in ResNet 4.0 database as of January 21, 2006, generated by MedScan technology version 1.8.2 after automatic curation described in [29].

## Discussion

### MedScan performance for automatic GO annotation

We used the hierarchical evaluating measure (HM) suggested in [33] to compare different annotation systems (see Methods for more detail). The shortcomings of a commonly-used alternative, the lowest common ancestor measure [34], are summarized in [33]. A thorough inspection of the similarities between various GO terms shows that the HM score appears to be strong and biologically meaningful when it is above 0.7. A similarity score below 0.4 is almost biologically meaningless. Thus, the similarity of 0.5, observed during comparison between the different public and MedScan annotations, represents a relatively weak correlation between two annotations. This result was unexpected, given the apparent 90% accuracy of protein-GO associations. To investigate the reasons for the moderate level of similarity between the extracted and public annotations, we identified GO groups that were systematically mis-assigned between two annotations. To identify these groups, we calculated the average similarity of every GO group in the automatically extracted annotation to its closest counterpart in public annotation. We found that GO terms which correspond to a specific cellular process have the highest similarity between annotations, while the frequently mis-assigned terms correspond to either items deleted from the MedScan GO dictionary or generic GO terms describing high-level signal transduction or transcriptional regulation processes, such as "protein amino acid dephosphorylation," "adenylate cyclase activation," etc. These high-level functions are rarely described directly in scientific literature, but can be inferred from molecular classification of a protein. For example, "regulation of transcription," "regulation of transcription, DNA-dependent," or "positive regulation of transcription from polymerase II promoter" can be assigned to all transcription factors. These annotations do not specify the biological process affected by a protein. Therefore, we conclude that GO annotation extracted by MedScan is biased towards proteins functions associated with more specific cellular processes.

The limited similarities between different GO annotations were documented at the BioCreAtIvE inter-annotator experiment (Critical Assessment of Information Extraction systems in Biology) [35]. Their results showed that there was a 39% chance of curators interpreting the text exactly and selecting the same GO term, a 43% chance that they extract a term from new/different lineage, and a 19% chance that they annotate a term from the same GO lineage. Our evaluation of the GO annotation by MedScan is equivalent to the BioCreAtIvE task 2.2 [28]. The NLP methods evaluated by BioCreAtIvE showed on average a 14% recall rate when compared with the manual annotation. The marked difference between MedScan and the NLP methods evaluated by BioCreAtIvE can be explained by the following cumulative reasons:

1) Automatic extraction methods are developed by teams of scientists who have their own standards in regards to how to interpret statements in scientific texts for GO annotation. Since the inter-annotator agreement test revealed only a 40% concordance between two human annotators, the 14% recall rate can be adjusted upwards by the inter-annotator accuracy. This adjustment makes the actual accuracy of NLP methods closer to 22%.

2) In our case, the human annotator was the developer of the NLP algorithm for the GO annotation. Therefore, the concordance between MedScan results and human evaluation is much closer. The MedScan GO annotation recovery rate adjusted downwards by the results of BioCreAtIvE inter-annotator agreement experiment is about 31%. We anticipate that a group of independent curators will report this rate upon inspection of MedScan results. This downward correction makes MedScan recovery rates closer but still superior to other NLP methods.

In light of the 40% concordance in the BioCreAtIvE inter-annotator experiments, one should not expect that the objective recall of any NLP method including MedScan will exceed 40% as well if measured against a the same literature corpus. We have confirmed this estimate by comparing MedScan performance with the performance of Gene Ontology curators from EBI. We found 28% overlap (317 out of 1,138) between protein-GO associations extracted by MedScan and associations found by human curators for the same set of GO groups in the same articles. We also showed that human curators miss at least 10% of the true facts described in the articles (137 out of 1,138).

Speed is a major advantage of the NLP methods. They can process a vast amount of literature that cannot be read by human annotators in a timely way. Our experience with MedScan shows that the estimates based on the processing of a limited number of articles can significantly underestimate the "global" recovery rate of the NLP technology. This happens due to the high redundancy of scientific literature that helps to overcome the incomplete "local" recovery rates [29]. A statement about a protein-GO association can be missed in the first article, but has a strong chance to be recovered from other articles that express the same fact in different linguistic forms. We showed that even on the relatively small literature corpus the MedScan recall rate was four times higher than that of human curators (4,649 annotations vs. 1,138 annotations). It exceeded manual curation recall rate even taking into account a reasonable correction for MedScan accuracy. Intrigued by the apparent superior performance of MedScan to human curators on the same literature corpus we have manually inspected the relations extracted by MedScan and missed by the human curators. We found that human curators tend to ignore sentences indirectly implying involvement of a protein in a cell process or where a protein-GO association is not the major statement, but is mentioned as an auxiliary fact. Additionally, the sentences describing the changes in expression level of a protein during a cell process were mostly ignored by human curators, while MedScan has interpreted them as true statements. Human curators also missed a lot of facts describing very generic cell processes such as "cell proliferation", "apoptosis" or "cell differentiation". During this evaluation we also found that MedScan tends to interpret some hypothetical, ambiguous or intent statements as a true statement about protein-GO association. Such statements usually summarize the findings of a paper in the discussion section and are highly important for comprehensive automatic extraction.

One more possible reason for superior MedScan performance over human curation is MedScan's protein names dictionaries. The efficiency of extracting protein-GO association depends not only on the correct recognition of GO terms in the text but also on the efficient protein name recognition. MedScan benefits from the four years of development that have produced highly accurate and comprehensive dictionaries of protein names and aliases. The MedScan performance to recognize and assign protein names far exceeds capabilities of any human curator because its dictionaries are manually curated and contain hundreds of thousands of protein names synonyms [36]. A human curator is incapable of memorizing all possible protein names and must use additional automated reference source to verify and assign protein names. Thus, if the computerized protein names resource available to human curators is not comprehensive enough they may not find a protein and disregard the protein-GO association. Because of this deficiency human curator tend to keep focus only on the main protein or main set of proteins described in a paper while ignoring statements about miscellaneous proteins that were made in support of the main conclusion of the article. MedScan extraction completely lacks this disadvantage and will pick up any statement from any section of the paper about any protein as long as the statement clearly points in the correct grammatical form to the involvement of a protein in a biological process.

The maturity of MedScan technology also explains its apparent superiority over other methods for automatic extraction (Figure 2). All six methods evaluated in BioCreAtIvE task 2.2 [28] relied on the statistical approach for fact extraction. The superior accuracy of the full-sentence parsing over statistical approach is well documented [24,40]. The recovery rate of the full-sentence semantic parsing is usually 2-3 times lower than in statistical methods. MedScan, however, has the best reported recovery rate for extracting protein-protein interactions among other full-sentence parsing NLP approaches [24]. This superiority is attributed to the quality of protein name dictionaries as well as to the developed ontology of linguistic patterns used by MedScan. While the set of linguistic patterns for extracting protein-GO associations is new and was developed for this work, we relied on existing MedScan dictionaries and algorithms for protein name recognition. Most likely, MedScan has outperformed other automated methods for extracting protein-GO association for the same two reasons: use of full-sentence semantic parsing and mature protein name dictionaries.

### Correlation of network clustering with functional modularity

Currently, the correlation between protein annotation and network clustering was demonstrated only for the physical interaction networks generated by high-throughput two-hybrid screens and mass-spectrometry experiments [2,37,38]. We have shown that a high degree of correlation exists between functional Gene Ontology annotation and clusters in both physical and regulatory networks automatically extracted from scientific literature by the NLP technology. The correlation was detected in both "directions": GO pathways had a higher than randomly expected link density, and densely linked network clusters significantly overlapped with GO groups.

The correlation between physical interaction clusters and GO groups was observed for all three branches of GO classification: biological processes, cellular component, and molecular function. The cellular component annotation provided the highest correlation. This suggests that the integrity of cellular organelles and complexes described by this GO branch, in addition to the specific "structural" interactions that define organelle topology, is maintained by the increased density of protein-protein interactions within an organelle. The network clustering within biological processes groups supports the view that the existence of functional modularity in a cell is primarily maintained by means of protein-protein physical interactions. We demonstrate this correlation by using both the complete GO classification as well as solely the annotation automatically extracted from the PubMed database. The Molecular function GO classification has the smallest number of densely linked GO groups: 54% in the physical interaction network and 44% in the *Regulation *network. Such a low correlation for the Molecular function classification is expected since this type of annotation does not relate to cellular processes and is based purely on protein sequence similarity. Still, the observed non-negligible correlation indicates that homologous proteins tend to participate in similar biological processes. Additionally, it may be due to the fact that protein paralogs tend to interact with each other more often than the evolutionary non-related proteins [39].

We found that clusters in all regulatory networks have a smaller correlation with GOA than the physical interaction clusters. Thus, it appears that the functional modules exchange regulatory signals between each other almost as often as signals propagate inside the functional modules themselves. Faster signal propagation within a module, as compared to the signaling between modules, is the only apparent reason of having functional clusters or modules in the physical interaction network without regulatory clustering. This ensures that an execution of a function happens first within the module and then the higher-level information exchange between modules takes place. The information exchange between modules can be viewed as an integration of signals from several processes by the cell.

### Biological significance of pathways built from GO annotation

Proteins in a pathway built from a Biological process GO annotation are involved in the same cellular process and are linked to each other by physical interactions and regulatory relations that are described in the literature and therefore have been thoroughly measured by dedicated experiments. Even though some of these relations can be conditional upon time, tissue and cellular localization, the majority of the interactions occur at all times and therefore represent the plausible mechanism for the regulatory and physical interaction events that mediate corresponding biological process. The fact that proteins in a pathway both belong to the same GO group and interact with each other increases the confidence of both GO annotation and protein-protein relations in a pathway. Generally, a GOA pathway contains fewer proteins than the corresponding GO group as it includes only the proteins linked into a connected network. Therefore, annotation of the proteins included into a pathway can be considered of a higher confidence than the annotation of the proteins that were left outside due to the absence of interactions.

The automatically constructed pathways can be used directly for the analysis of high-throughput data such as microarray gene expression. That in principle could produce results different from the standard GO group analysis and of a higher confidence. In addition, GOA pathways can be used as initial data for further manual curation, significantly simplifying this procedure. Similar GO pathways can be built for other type of protein annotations such as Protein Disease Ontology, Gene Ontology for other organisms, or proteins identified in whole genome genetic association studies.

## Conclusion

The GO annotation extracted by the NLP technology described here expands and enriches the existing manual GO annotation. Two principal attributes of a protein's cellular role, its functional annotation and its place in the topology of a cellular protein-protein network, are significantly related to each other and, therefore, should complement each other when used for the interpretation of high-throughput experiments. The GO functional modularity correlates most strongly with the clustering in the physical interaction network, which is another manifestation of the fact that any biological process requires a certain supporting structural molecular organization in a cell.

In future, we plan to improve the precision and recall rates of the GO extraction by NLP by thoroughly analyzing the cases of its incorrect interpretation or ignoring of the relevant parts of the text. Redundancy of scientific literature and its effect on global NLP performance is another possible topic of our future research. We also plan to use the correlation between the GOA and protein network clustering to improve the quality of both the GO and protein interaction datasets by automatically rectifying strong mutual contradictions.

## Methods

### Preparation of the GO dictionary for MedScan

As a first step, we constructed the dictionary of GO term descriptors suitable for efficient identification in text. We narrowed the set of terms from the "Biological processes" of GO to a reasonably informative and consistent subset of cellular-level biological processes by removing some terms that we considered inappropriate, too general, or too detailed. In particular, the following terms were removed from the dictionary:

• GO terms representing positive or negative regulation of another GO term; GO terms referencing individual proteins; GO terms representing organism-level behavioral processes

• GO terms representing steps of protein metabolism

• High-level GO terms with high frequency of occurrence and low informational content (e.g., "transcription", "transport", "development")

• GO terms for highly specific biochemical processes.

In addition, we preprocessed the GO terms containing chemical names using the MedScan preprocessing algorithm for tagging chemical names in text [40]. Identified names were replaced with numerical identifiers unique for each chemical substance. This replacement allowed us to detect variations of GO terms containing synonyms of chemical names in the text and point them to the original GO term.

Similarly, we have created a small dictionary of word synonyms, containing names of tissues and cell types, frequently used names of cellular components, and notations of high-level processes often used in GO terms, e.g., transport, synthesis/degradation, movement, and assembly/disassembly. Finally, we have manually added commonly used synonyms to our dictionary of GO term descriptors. The synonyms were taken from the existing MedScan dictionaries as well as by adding adjective forms of the noun synonyms such as "mitosis-mitotic," for example. In the end, the dictionary contained 6,318 descriptors representing 5,764 unique GO terms from the "biological process" category.

### Detection of GO terms in scientific text

Our detection of GO terms in the scientific text involves the following steps.

**1. **Input text is scanned for the names of mammalian proteins [36] and chemical substances and all found substrings are tagged with corresponding numerical identifiers.

**2. **The GO term dictionary is loaded. During loading, it is normalized in a manner described in step 5 of this process, and each word in each GO term descriptor is replaced with the corresponding numerical identifier. This identifier is first searched for in the synonym dictionaries for chemicals, tissues, cell types, and compartments. If a word is not found in the synonym dictionaries, a new identifier is assigned to each unique word. The main purpose of this step is to normalize terminological variations in text.

**3. **Input text is split into individual sentences, and each word in a sentence is replaced with the corresponding numerical identifier the same way as it is done for the GO term dictionary in the previous step. The algorithm splitting text into sentences uses the following token as a separator: a period followed by any number of whitespaces and then a capital letter.

**4. **The minimal noun phrases (MNPs) are detected in every sentence, using regular expression over the syntactic categories assigned by the dedicated Part of Speech (POS)-tagging program. We used the following regular expression for MNP detection:

(DET|Pron|D)* ((COMMA|AND|OR|BUT_NOT)? J)* N N*

where **DET **is determiner, **Pron **is pronoun, **J **is adjective, **D **is adverb, and **N **is noun. Other symbols are introduced in Additional file 1.

**5. **MNPs separated by prepositions are normalized. The main purpose of this step is to disregard prepositional variations in a noun phrase structure. Normalization is done by flipping the order of MNPs and removing prepositions separating MNPs. The MNP order is changed so the phrase "*MNP1 preposition MNP2 preposition MNP3*" becomes "*MNP3 MNP2 MNP1*". For example:

*"rapid ****de-differentiation ****of ****hepatocytes ****from human ****liver ****into fat ****cells***"

is normalized into

*"fat ****cells ****human ****liver hepatocyte ****rapid ****de-differentiation***"

During the normalization, the information about "head" word from each MNP is preserved. In the preceding example, the words "**de-differentiation**", "**liver**", "**hepatocytes" **and "**cells**" are marked as head words.

**6. **Finally, normalized GO term descriptors are linearly matched over a noun phrase by sequentially comparing the numerical identifier of each word in a GO descriptor with numerical identifiers of words in the normalized noun phrase. A successful match is allowed to contain any number of gaps between the words in the GO descriptor, but all words of the descriptor have to be matched. In addition, the following two constraints must be satisfied:

• The entire match must occur within the borders of a single MNP, or

• If a match spans more than one MNP within a normalized NP, the "head" words of all spanned minimal NPs must be matched.

Initially, all possible matches of different GO terms within each normalized NP are considered and each match is assigned a simple score: S = L - G - H, where *L *is the number of words in a GO term, *G *is the total length of gaps between GO term words, and *H *is the distance (in words) from the end of a match to the "head" of a normalized noun phrase. In the end, the set of the highest-scoring non-intersecting matches is selected and matches in the text are tagged with the corresponding GO identifier.

### Automatic extraction of protein associations with a GO term

Associations between proteins and GO terms are extracted using a small set of linguistic patterns (defined using regular expression-like notation) matched over a linear sequence of sentence words. The matching is done by automatically constructed deterministic finite automaton (DFA), which will be described in detail elsewhere. Following are some of its features that are relevant for GO annotation. First, our DFA recognizes protein names and GO terms tagged in the text by the MedScan preprocessor and treats them as variables. Second, it can match words in all grammatical forms using morphological word-form reduction [41]. The DFA runs over individual input sentences and attempts to match all regular expression patterns in Additional file 1. Once a match is encountered, the corresponding relation between two pattern variables is extracted.

### Pairwise comparison of different GO annotations

To compare public and automatically extracted annotations, we chose the method described in Kiritchenko *et al. *[33]. Briefly, each GO term is augmented with all of its parents along all possible paths to this term from the root of the ontology. (The root node itself is not included.) When comparing GO terms, two metrics are introduced: K1 and K2. If a protein is annotated differently in two annotations (i.e., it belongs to two different GO groups) K1 is equal to the number of the common parental nodes between two GO groups divided by the total number of parental nodes for the first group. K2 is equal to the number of the common parental nodes between two GO groups divided by the total number of parental nodes for the second group. The local similarity between two GO terms is then calculated as F-score: 2*K1*K2/(K1+K2)).

Given the local similarity between two GO terms, we can define the asymmetric similarity between two GO annotations. Let the first annotation **A **contains terms {A_1_, A_2_, A_3_... A_n_} terms and the second annotation **B **for the same protein contains terms {B_1_, B_2_, B_3_... B_m_}. The similarity between two annotations can be defined as an average similarity among N pairs of GO terms, where each pair contains one term from the set {A_1_, A_2_, A_3_... A_n_} and its most similar GO term among B_1_, B_2_, B_3_... B_m_:

SIM(A->B)  =∑iSIM(maxj{AiBj})N
 MathType@MTEF@5@5@+=feaafiart1ev1aaatCvAUfKttLearuWrP9MDH5MBPbIqV92AaeXatLxBI9gBaebbnrfifHhDYfgasaacH8akY=wiFfYdH8Gipec8Eeeu0xXdbba9frFj0=OqFfea0dXdd9vqai=hGuQ8kuc9pgc9s8qqaq=dirpe0xb9q8qiLsFr0=vr0=vr0dc8meaabaqaciaacaGaaeqabaqabeGadaaakeaabaGaee4uamLaeeysaKKaeeyta0KaemikaGIaeeyqaeKaeeyla0IaemOpa4JaemOqaiKaemykaKIaeeiiaaIaeeiiaaIaemypa0ZaaSaaaeaadaaeqbqaaiabdofatjabdMeajjabd2eanjabcIcaOiabb2gaTjabbggaHjabbIha4naaBaaaleaacqWGQbGAaeqaaaqaaiabdMgaPbqab0GaeyyeIuoakmaacmaabaGaemyqae0aaSbaaSqaaiabdMgaPbqabaGccqWGcbGqdaWgaaWcbaGaemOAaOgabeaaaOGaay5Eaiaaw2haaiabcMcaPaqaaiabd6eaobaaaaaa@4F3D@

Finally, we define the global similarity between two annotations sharing many common but differently annotated proteins as an average similarity between two alternative sets of GO terms from every protein present in both annotations.

### ResNet database

The protein networks used in this work were extracted from the ResNet 4.0 database of mammalian protein relations, described in detail in [29]. The number of proteins and the number of most commonly used interactions accumulated in ResNet 4.0 are shown in Table 5. The database is constructed from relations extracted automatically by the full-sentence parsing NLP technology called MedScan [24]. MedScan extracts about 1,000,000 relations between mammalian proteins, chemicals and protein functional classes from the entire Medline and 43 full-text journals. The extracted facts are automatically curated as described previously [29]. It takes the MedScan technology three days to processes the entire Medline on a regular personal computer. The ResNet database is re-generated every year, following the change of the baseline in Medline database.

### Construction of biological process pathways

The biological process pathways were built by connecting all proteins that belong to the same GO group with relations from the ResNet 4.0 database. All proteins annotated by the child GO group were included into the parent GO group recursively for all child groups. Types of relations between proteins and their abundance in ResNet 4.0 are listed in Table 5. In the first step, proteins of the putative pathway (GO group) were connected by physical interactions. The remaining unconnected proteins in the group were linked to the emerging pathway by indirect regulatory relations such as *MolTransport*-indicating regulation of molecular translocation, *Expression *and *PromoterBinding*-both indicating regulation of a target via gene expression; and *Regulation*-indicating the regulatory event of an unknown mechanism. If a protein could not be connected to any protein in the pathway by any of those relations, such protein was removed from the pathway at the end of the algorithm run. This order of connecting proteins does not effect the protein selection and was designed to give a priority to direct physical interactions over indirect regulatory relations for labeling links with dual identity. A collection of automatically generated pathways for the GO Biological Processes annotation is freely available from the Ariadne Genomics Webpage and it can be accessed by downloading the free demo version of Pathway Studio software.

### Finding network communities

To find clusters in the physical interaction network, we used annealing of the network-based ferromagnetic Potts model with an adjustable antiferromagnetic term [31]. This clustering method is a generalization of the approach suggested in [32] that allows more flexibility in defining the size and link density of the network communities we wish to determine. A variable (often called *spin*) is assigned to each network vertex; linked pairs of vertices with spins in the same state are energetically favored. Initially, spins are assigned at random, the number of possible spin states **q **is usually of order of the number of vertices *N*, we used q = N/5. The model is allowed to evolve by changing spins of randomly selected vertices according to the Metropolis algorithm with a gradually lowered temperature. Usually, each vertex is allowed to sample all **q **spin states several times after which the temperature is decreased by a small fraction (1%). To prevent condensation of all vertices into the same state, an antiferromagnetic energy penalty that depends on the number of vertices *n*_*i *_in each state state is introduced H'=γ∑i=1qniα+1
 MathType@MTEF@5@5@+=feaafiart1ev1aaatCvAUfKttLearuWrP9MDH5MBPbIqV92AaeXatLxBI9gBaebbnrfifHhDYfgasaacH8akY=wiFfYdH8Gipec8Eeeu0xXdbba9frFj0=OqFfea0dXdd9vqai=hGuQ8kuc9pgc9s8qqaq=dirpe0xb9q8qiLsFr0=vr0=vr0dc8meaabaqaciaacaGaaeqabaqabeGadaaakeaacqWGibascqGGNaWjcqGH9aqpiiGacqWFZoWzdaaeWbqaaiabd6gaUnaaDaaaleaacqWGPbqAaeaacqWFXoqycqGHRaWkcqGHXaqmaaaabaGaemyAaKMaeyypa0JaeyymaedabaGaemyCaehaniabggHiLdaaaa@3E97@[31]. An aligned link defines the unit of energy, that is, two linked vertices in the same spin state contribute -1 to the total energy of the system. For finding communities in the ResNet 4.0 protein-protein binding network we used γ = 0.005–0.02 and α = 1 – 1.5.

When the evolution stops at a sufficiently low temperature, linked vertices in the same state are declared network communities. This method allows one to find an *a priori *unknown number of possibly overlapping mesoscopic clusters in a sparse network with a low link density contrast.

## Authors' contributions

ND designed and implemented algorithm for protein annotation using MedScan technology and for statistical comparison of different GOA,

AY developed an algorithm for pathway building using GO groups and wrote the manuscript,

SE implemented algorithm for protein annotation using MedScan technology,

IM was the general manager and contributed to the manuscript,

II developed the modification of Potts model clustering method, preformed statistical evaluation of the correlation between GOAs and network clustering and wrote the manuscript.

All authors have read and approved the final manuscript.

## Supplementary Material

Additional file 1 Regular expression patterns used for automatic extraction of protein associations with GO terms by MedScan deterministic finite automaton (DFA). The file contains the complete list of linguistic patterns used for extraction of GO annotation by MedScan. Click here for file
