# Supplementary material for: Automatic extraction of gene ontology annotation and its correlation with clusters in protein networks
Source: BMC Bioinformatics. 2007 Jul 10;8:243. doi: 10.1186/1471-2105-8-243 (PMC1940026; doi:10.1186/1471-2105-8-243)
Supplement: Additional file 1 — Regular expression patterns used for automatic extraction of protein associations with GO terms by MedScan deterministic finite automaton (DFA). The file contains the complete list of linguistic patterns used for extraction of GO annotation by MedScan. [file 1471-2105-8-243-S1.doc]

### Additional file 1. Regular expression patterns used for automatic extraction of protein associations with GO terms by MedScan deterministic finite automaton (DFA).

Each pattern is a sequence of elements with optional operators controlling their occurrence in a given position. Pattern elements are individual words or sets of words (word sets), named variables, other sub-patterns, or special symbols. During the matching process, they are matched against individual words of a sentence to find all acceptable occurrences of the pattern over the sentence. A word set is a list of words allowed at a given position in a sentence. Word sets can also include negation symbol **^** in the beginning, which indicates that any word except for ones specified in the word-set can occur at the given position. A match between pattern and sentence words is allowed in any grammatical form. For example, two most frequent variations include singular/plural form for nouns and inflectional variation for verbs. Such word form recognition is indicated by the symbol **~** following a word in the pattern. Named variables are denoted as %*name* and are used to match tagged named entities: proteins and GO terms. Note, that each pattern always has two variables: %Protein and %CellProcess. The table below provides a more detailed explanation of pattern syntax elements, along with word sets and sub-patterns used in our extraction patterns. A list of patterns follows:

**%Protein @NO_NEG ($POS_REGULATE|$NEG_REGULATE|$UNK_REGULATE|$CAUSE|$HAS_FUNCTION_V_AS|$CP_SPECIALS|@PLAY_ROLE) @.* %CellProcess**

**%CellProcess @NO_NEG ($POS_REGULATE|$NEG_REGULATE|$UNK_REGULATE|$CAUSE|$HAS_FUNCTION_V_AS|$CP_SPECIALS) [by through] @.* %Protein**

**%CellProcess @NO_NEG (requires|(depend~ on)) @.* %Protein**

**%Protein @NO_NEG ($BE|$HAVE_V|$IS_V|$HAS_FUNCTION_V_AS|@APPOS) [^$NEG]* ($POS_REGULATION|$POS_REGULATOR|$NEG_REGULATION|$NEG_REGULATOR|$UNK_REGULATION|$UNK_REGULATOR|$IMPORTANT) @.* %CellProcess**

**%Protein @NO_NEG ($BE|$HAVE_V|$IS_V|$HAS_FUNCTION_V_AS|@APPOS) [^$NEG]* %CellProcess ($POS_REGULATION|$POS_REGULATOR|$NEG_REGULATION|$NEG_REGULATOR|$UNK_REGULATION|$UNK_REGULATOR)**

**(($ABILITY_N of @N_ATTR %Protein)|(%Protein @N_ATTR $ABILITY_N)) [on to in] @.{0,5} %CellProcess**

**((($POS_REGULATION|$NEG_REGULATION|$NEG_REGULATION) of @N_ATTR %CellProcess)|(%CellProcess @N_ATTR ($POS_REGULATION|$NEG_REGULATION|$UNK_REGULATION))) by @.{0,5} %Protein**

%CellProcess protein~? %Protein

| **Notation and elements used in extraction patterns**. | | |  |  |
| --- | --- | --- | --- | --- |
| **Notation** | **Description** | **Examples** | | |
| **Pattern elements** | | | | |
| **$[***word word* **… ]** | Set of words allowable at pattern position |  | | |
| ***** | Word match repeated 0 or more times |  | | |
| **?** | Optional word match |  | | |
| **~** | Matches word in all its grammatical forms |  | | |
| **%** | Output variable |  | | |
| **()** | Pattern grouping |  | | |
| **@.** | Matches any word |  | | |
| **{n,m}** | Word match repeated in a specified range of times |  | | |
| **@***name* | Name of sub-pattern |  | | |
| **|** | Logical OR |  | | |
| **[^ ]** | Set of words prohibited at pattern position |  | | |
| **Word sets** | | | | |
| **$BE** | All forms of words “is”, “be”, “do”,“will” | is, are, be, being, was, do, did, is | | |
| **$J** | Adjective | small, purified, human | | |
| **$DET** | Determiners | a, an, the, this, these, those | | |
| **$D** | Adverbs | well, also, specifically | | |
| **$NEG** | Negations | no, not, never, neither, nor | | |
| **$POS_REGULATE** | Verb meaning positive regulation | activate, induce, enhance | | |
| **$NEG_REGULATE** | Verb meaning negative regulation | block, inhibit, preclude | | |
| **$UNK_REGULATE** | Verb meaning unknown regulation | regulate, modulate, alter | | |
| **$CAUSE** | Verbs meaning causative relation | cause, contribute, lead | | |
| **$CP_SPECIALS** | Small set of special “cell process” verbs | play, protect, catalyze | | |
| **$HAVE_V** | Verbs indicating presence of property | show, display, reveal, bear | | |
| **$IS_V** | Verbs indicating being in/arriving at state | become, remain, became | | |
| **$HAS_FUNCTION_V_AS** | Verbs indicating presence of function | act, work ,function, serve | | |
| **$POS_REGULATION** | Nouns meaning positive regulation | activation, induction | | |
| **$POS_REGULATOR** | Nouns meaning positively-acting agent | activator, enhancer | | |
| **$NEG_REGULATION** | Nouns meaning negative regulation | blockade, inhibition, arrest | | |
| **$NEG_REGULATOR** | Nouns meaning negatively-acting agent | blocker, antagonist, inhibitor | | |
| **$UNK_REGULATION** | Nouns unknown positive regulation | modulation, regulation | | |
| **$UNK_REGULATOR** | Nouns meaning affecting agent | mediator, regulator | | |
| **$IMPORTANT** | Adjective indicating importance | critical, important, pivotal | | |
| **$ABILITY_N** | Noun indicating ability | ability, capability, role | | |
| **Sub-patterns** | | | | |
| **@N_ATTR** | Optional modifiers preceding noun phrase | **$DET* $D* $J*** | | |
| **@APPOS** | Start of an apposition | **',' [a an]** | | |
| **@NO_NEG** | Absence of negation | **[^ $NEG fail~]*** | | |
| **@PLAY_ROLE** | “play role” sub-pattern | **play~ $DET* $D* $J* role in** | | |
